# Supplementary material for: Median Nerve Stimulation for Treatment of Tics: Randomized, Controlled, Crossover Trial
Source: J Clin Med. 2023 Mar 27;12(7):2514. doi: 10.3390/jcm12072514 (PMC10095326; doi:10.3390/jcm12072514)
Supplement: Supplementary file 1 [file jcm-12-02514-s001.zip › Supplement/jcm-2267981-supplementaryAIedits.docx]

# Material for online supplement

Contents:

- Clinical Global Impression–Efficacy Index (participant)
- Comments on improvement with stimulation from visit debriefing
- Supplementary Figure 1: Tic Frequency before and after stimulation ends
- CONSORT Checklist for Randomized Crossover Trials
- Video clips from 3 participants who reported substantial benefit from MNS

## Section S1. Clinical Global Impression–Efficacy Index (participant)

Benefits compared to side effects for rhythmic and arrhythmic stimulation. Table entries below indicate number of participants whose ratings at the end of the study day were those given in the row and column headings.

### Rhythmic stimulation


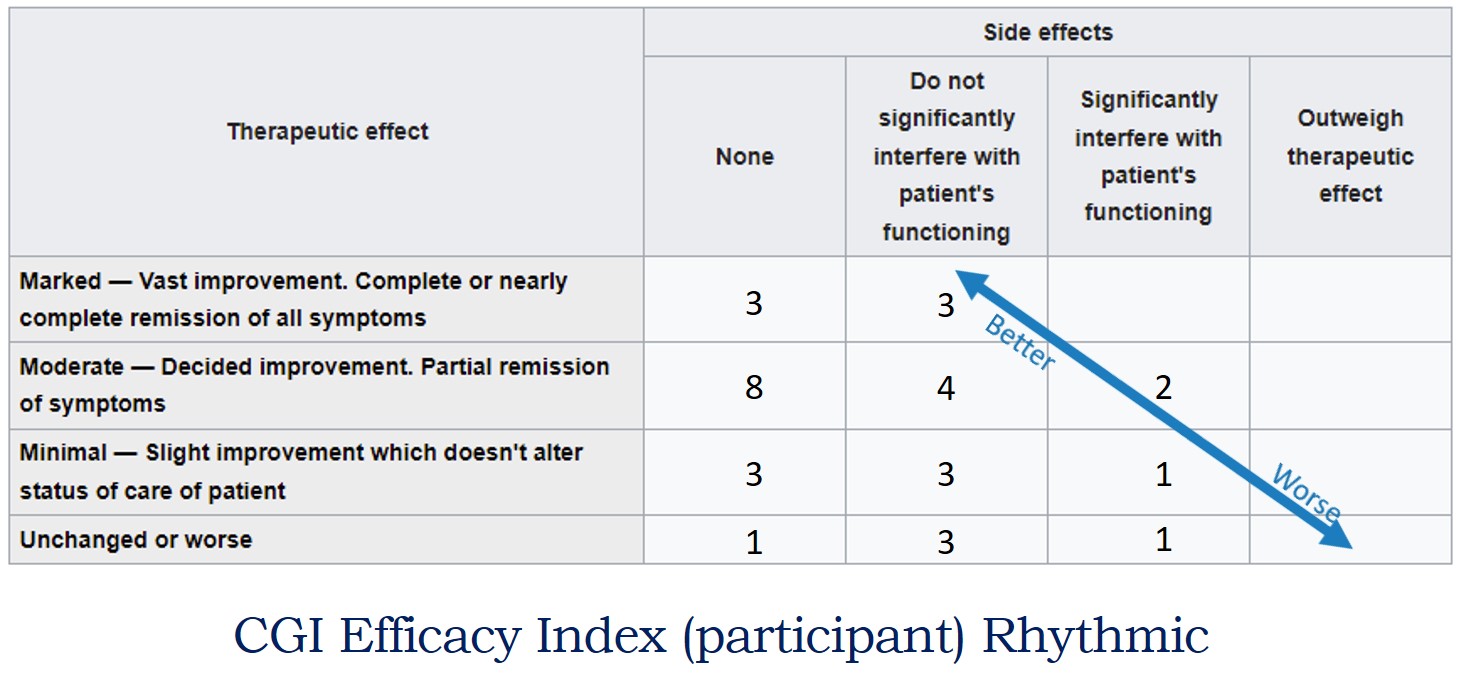


### Arrhythmic stimulation


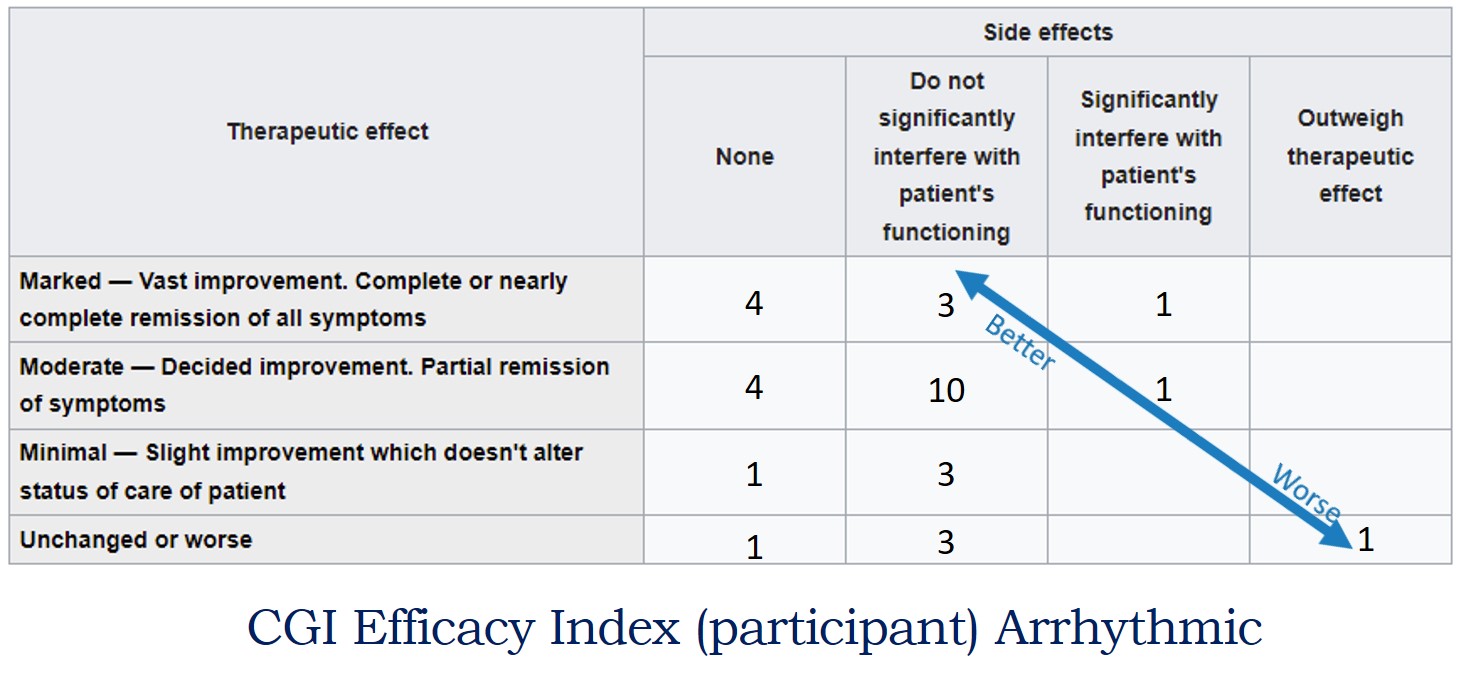


## Section S2. Comments on improvement with stimulation from visit debriefing

### Visit 1 (Received Rhythmic Stimulation)

| Less premonitory urges, tics felt habitual instead of compulsive |
| --- |
| I can’t decide if being aware, knowing the stimulation is on, if I only thought I was improving or if I was really improving. |
| Fairly decreased frequency of tics. |

### Visit 2 (Received Rhythmic Stimulation)

| Urge decreased a bit, not as much improvement as last time |
| --- |
| Felt some decrease in tics but did not feel the relief from tics |
| “My [tilt head to] side tic was much worse today.” Clarifies this means the whole time, stimulation on or stimulation off. |
| Perhaps the distraction of stimulation affected my ticcing, I’m not sure. |
| A sense of “at ease,” just being at ease with myself. |

### Visit 1 (Received Arrhythmic Stimulation)

| Felt more at ease, less feelings of jittery |
| --- |
| She felt that the electrical stimulation felt different on each stimulation block, like one was more steady, others were more pulsating, one was more tingling. |
| “It was hard to tell at first, but I noticed that over time I had less tics when the stimulation was on than when it wasn’t.” |
| Urge decreased; distracted from tics by stimulation. |
| Brief calm without an urge to tic. |
| The moment it turned on was worse—surprise. But by the end, it felt like tics would have been much easier to suppress if I were trying to suppress them, and when I did tic, it didn’t feel like it needed to be as violent or severe. |

### Visit 2 (Received Arrhythmic Stimulation)

| Equal benefit as at other visit |
| --- |
| Seem much more relaxed and much less dependent on having to satisfy a tic. |
| Benefit lasted longer than at visit 1. |
| No improvement today |
| When stimulation ends there’s a sense of relief that it’s over. |
| I felt more still. Calm. |

## Supplementary Figure S1: Tic Frequency before and after stimulation ends

| 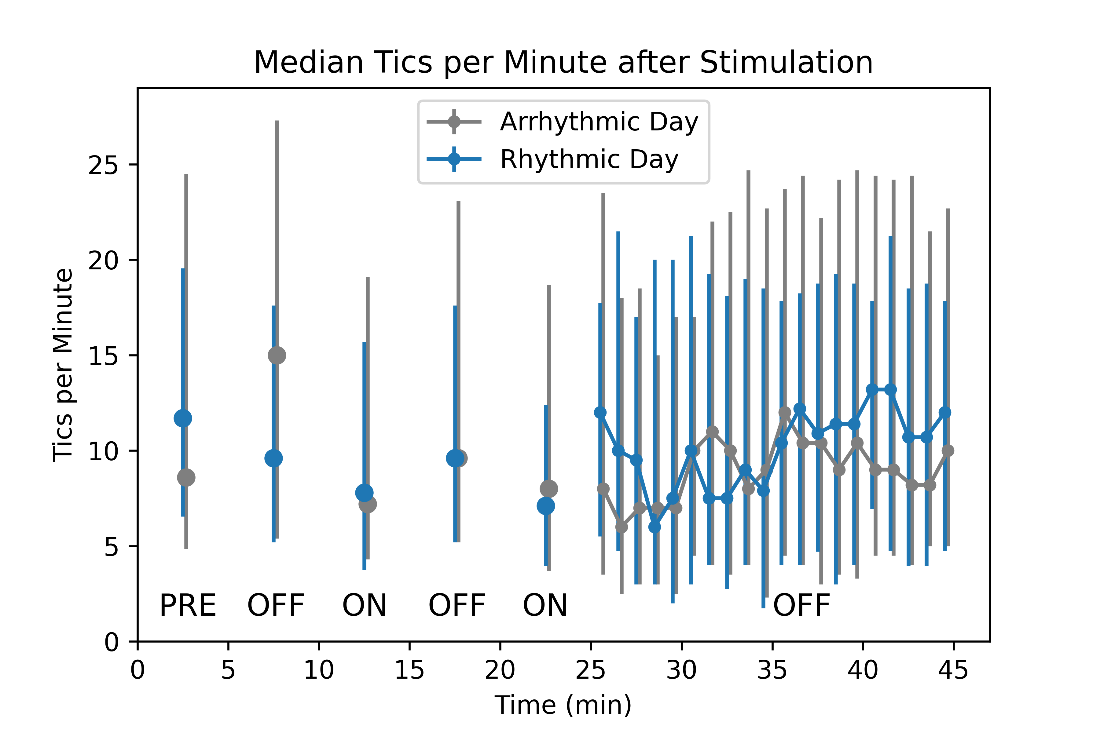 ***Figure S1 Supplement.* Tic frequency before and after stimulation ends.** Median tic frequency is shown for all 5-minute blocks (see Figure 1). Block 0 (5-minute baseline OFF block before stimulation, labeled “PRE”) and Blocks 5-8 (5-minute blocks OFF / ON / OFF / ON after the four 1-minute blocks) are plotted at times 2.5, 7.5, 12.5, 17.5 and 22.5 minutes. Median tic frequency for each of the 20 minutes after the last stimulation ON block (i.e., from Blocks 9-12) is plotted at times 25.5-44.5 min. The last 15 minutes include LOCF data as described in Methods. Vertical bars represent 25th and 75th percentiles. |
| --- |

Section S3. **
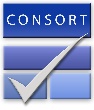
CONSORT 2010 checklist of information to include when reporting a randomised trial***

| **Section/Topic** | **Item No** | **Checklist item** | **Reported on page No** |
| --- | --- | --- | --- |
| **Title and abstract** | | | |
|  | 1a | Identification as a randomised crossover trial in the title | 1 |
|  | 1b | Specify a crossover design and report all information outlined in table 2 | 1 |
| **Introduction** | | | |
| Background and objectives | 2a | Scientific background and explanation of rationale | 2 |
|  | 2b | Specific objectives or hypotheses | 2 |
| **Methods** | | | |
| Trial design | 3a | Rationale for a crossover design. Description of the design features including allocation ratio, especially the number and duration of periods, duration of washout period, and consideration of carry over effect | 3-4 |
|  | 3b | Important changes to methods after trial commencement (such as eligibility criteria), with reasons | 5-6 |
| Participants | 4a | Eligibility criteria for participants | 2 |
|  | 4b | Settings and locations where the data were collected | 3 |
| Interventions | 5 | The interventions with sufficient details to allow replication, including how and when they were actually administered | 3-4 |
| Outcomes | 6a | Completely defined pre-specified primary and secondary outcome measures, including how and when they were assessed | 5-6 |
|  | 6b | Any changes to trial outcomes after the trial commenced, with reasons | 5 |
| Sample size | 7a | How sample size was determined, accounting for within participant variability | 2 |
|  | 7b | When applicable, explanation of any interim analyses and stopping guidelines | N/A |
| Randomisation: |  |  |  |
| Sequence generation | 8a | Method used to generate the random allocation sequence | 3 |
|  | 8b | Type of randomisation; details of any restriction (such as blocking and block size) | 3 |
| Allocation concealment mechanism | 9 | Mechanism used to implement the random allocation sequence (such as sequentially numbered containers), describing any steps taken to conceal the sequence until interventions were assigned | 3 |
| Implementation | 10 | Who generated the random allocation sequence,§ who enrolled participants, and who assigned participants to the sequence of interventions | 2-3 |
| Blinding | 11a | If done, who was blinded after assignment to interventions (for example, participants, care providers, those assessing outcomes) and how | 3 |
|  | 11b | If relevant, description of the similarity of interventions | 3 |
| Statistical methods | 12a | Statistical methods used to compare groups for primary and secondary outcomes which are appropriate for crossover design (that is, based on within participant comparison) | 5-6 |
|  | 12b | Methods for additional analyses, such as subgroup analyses and adjusted analyses | 6 |
| **Results** | | | |
| Participant flow (a diagram is strongly recommended) | 13a | The numbers of participants who were randomly assigned, received intended treatment, and were analysed for the primary outcome, separately for each sequence and period | 6-7 |
|  | 13b | No of participants excluded at each stage, with reasons, separately for each sequence and period | 6 |
| Recruitment | 14a | Dates defining the periods of recruitment and follow-up | 2 |
|  | 14b | Why the trial ended or was stopped | 2 |
| Baseline data | 15 | A table showing baseline demographic and clinical characteristics by sequence and period | 2 |
| Numbers analysed | 16 | Number of participants (denominator) included in each analysis and whether the analysis was by original assigned groups | 6 |
| Outcomes and estimation | 17a | For each primary and secondary outcome, results including estimated effect size and its precision (such as 95% confidence interval) should be based on within participant comparisons.¶ In addition, results for each intervention in each period are recommended | 6-12 |
|  | 17b | For binary outcomes, presentation of both absolute and relative effect sizes is recommended | N/A |
| Ancillary analyses | 18 | Results of any other analyses performed, including subgroup analyses and adjusted analyses, distinguishing pre-specified from exploratory | 6-12 |
| Harms | 19 | Describe all important harms or untended effects in a way that accounts for the design (for specific guidance see CONSORT for harms) | 11 |
| **Discussion** | | | |
| Limitations | 20 | Trial limitations, addressing sources of potential bias, imprecision, and, if relevant, multiplicity of analyses. Consider potential carry over effects | 13 |
| Generalisability | 21 | Generalisability (external validity, applicability) of the trial findings | 12-13 |
| Interpretation | 22 | Interpretation consistent with results, balancing benefits and harms, and considering other relevant evidence | 12-13 |
| **Other information** | | |  |
| Registration | 23 | Registration number and name of trial registry | 1 |
| Protocol | 24 | Where the full trial protocol can be accessed, if available | 3 |
| Funding | 25 | Sources of funding and other support (such as supply of drugs), role of funders | 13 |

CONSORT=Consolidated Standards of Reporting Trials.

- * Note: page numbers are optional depending on journal requirements.
- § Random sequence here refers to a list of random orders, typically generated through a computer program. This should not be confused with the sequence of interventions in a randomised crossover trial, for example receiving intervention A before B for an individual trial participant.
- ¶ A within participant comparison takes into account the correlation between measurements for each participant because they act as their own control, therefore measurements are not independent.

*We strongly recommend reading this statement in conjunction with the CONSORT 2010 Explanation and Elaboration for important clarifications on all the items. If relevant, we also recommend reading CONSORT extensions for cluster randomised trials, non-inferiority and equivalence trials, non-pharmacological treatments, herbal interventions, and pragmatic trials. Additional extensions are forthcoming: for those and for up to date references relevant to this checklist, see [www.consort-statement.org](http://www.consort-statement.org).

**Table S1 - Items to include when reporting a randomised crossover trial in a journal abstract**

| **Item** | **Description** |
| --- | --- |
| Authors | Contact details for the corresponding author |
| Trial design | Description of the trial design (crossover trial and number of periods) |
| Methods: |  |
| Participants | Eligibility criteria for participants and the settings where the data were collected |
| Interventions | Interventions intended for all participants |
| Objective | Specific objective or hypothesis |
| Outcome | Clearly defined primary outcome for this report |
| Randomisation | How participants were allocated to sequences |
| Blinding (masking) | Whether or not participants, care givers, and those assessing the outcomes were blinded to group assignment |
| Results: |  |
| Numbers randomised | Number of participants randomised to each sequence |
| Recruitment | Trial status |
| Numbers analysed | Number of participants analysed |
| Outcome | For the primary outcome, the estimated effect size and its precision based on within participant comparisons |
| Harms | Important adverse events or side effects |
| Conclusions | General interpretation of the results |
| Trial registration | Registration number and name of trial register |
| Funding | Source of funding |
