# Supplementary figures and images for: Median Nerve Stimulation for Treatment of Tics: Randomized, Controlled, Crossover Trial
Source: J Clin Med. 2023 Mar 27;12(7):2514. doi: 10.3390/jcm12072514 (PMC10095326; doi:10.3390/jcm12072514)

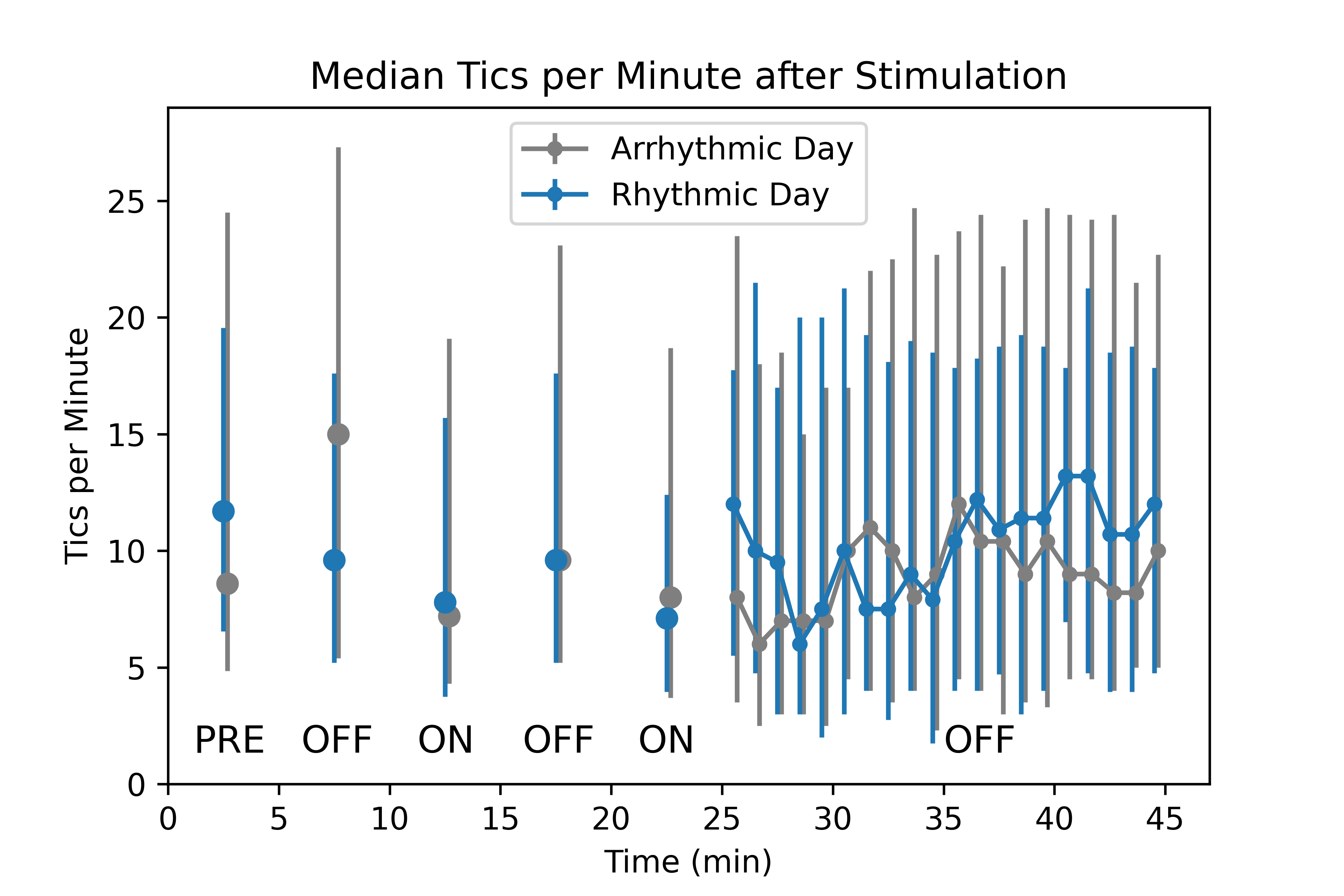

Supplement: Supplementary file 1 [file jcm-12-02514-s001.zip › Supplement/SupplementalFigure1.png]
